# Kupffer Cells Regulate iNKT Cells Through Il‐12 to Mitigate the Extent of APAP‐Induced Damage in the Liver

**DOI:** 10.1111/jcmm.70549

**Published:** 2025-07-02

**Authors:** Yinling Li, Shuang Wen, Jie Liu, Shuwei Li, Junping Shi, Haitao Wang

**Affiliations:** ^1^ Department of School of Life Sciences Zhejiang University of Traditional Chinese Medicine Zhejiang Hangzhou China; ^2^ Department of Translational Medicine Platform The Affiliated Hospital of Hangzhou Normal University Zhejiang Hangzhou China; ^3^ Department of Immunology Nanjing Medical University Nanjing Jiangsu China; ^4^ INSERM‐U1149, CNRS‐ERL8252, Centre de Recherche Sur L'inflammation (CRI), Laboratoire D'excellence Inflamex, Faculté de Médecine Xavier Bichat Université de Paris‐Cité Paris France; ^5^ Xinjiang Production and Construction Corps Key Laboratory of Protection and Utilization of Biological Resources in Tarim Basin, College of Life Science and Technology Tarim University Xinjiang China; ^6^ William Harvey Research Institute, Barts and the London School of Medicine and Dentistry Queen Mary University of London London UK

**Keywords:** adoptive transfer, iNKT cells, Kupffer cells, liver injury, neutrophils

## Abstract

To explore the innate immune response in the occurrence and development of APAP‐induced drug‐induced liver injury and the biological effect caused by the interaction between immune cells through real‐time, in vivo analysis. We used conventional molecular biology technology and multi‐photon confocal live imaging to explore the effect of the interactive dialogue between iNKT and Kupffer cells on neutrophil recruitment in the occurrence and development of APAP‐induced liver injury. iNKT cell deficient mice were more prone to liver injury induced by excessive APAP, and the degree of liver injury was more severe. After injury, iNKT cells were recruited into the liver and showed IL‐4 high expression activation mode. Furthermore, we also found that Kupffer cells in APAP induced liver injury produce M1 polarisation and highly expressed IL‐12, that Kupffer cells regulate the activation of iNKT cells through IL‐12; and that IL‐4 produced by iNKT cells in the liver can also inhibit inflammatory damage caused by neutrophil recruitment. Our study shows that Kupffer cells produce IL‐12 and promote activated iNKT cells to produce more IL‐4, and inhibit the recruitment of neutrophils, thereby reducing the degree of liver inflammatory injury induced by APAP.

## 1 Introduction

Acetaminophen (APAP) is a widely used nonsteroidal anti‐inflammatory drug (NSAID) around the world. Although it has positive antipyretic and analgesic effects, its drug dependence and liver injury or even liver failure caused by overdose remain a major problem we are facing [1]. APAP generates the active metabolite N‐acetyl‐p‐benzoquinone imine (NAPQI) via cytochrome P450 (CYP450) [2]. Excessive NAPQI will consume glutathione (GSH) and bind to macromolecules in hepatocytes, leading to mitochondrial dysfunction and production of reactive oxygen species (ROS) [3], eventually leading to hepatocyte necrosis, membrane permeability change, and release of toxic substances into the blood [4]. As the dominant organ of innate immunity, the liver contains a high proportion of innate immune cells. Although the mechanism of APAP‐induced liver injury (AILI) is fully understood, the role of immune cells, such as NKT cells and Kuffer cells in AILI remains unclear.

Natural killer T (NKT) cells are a special subset of T lymphocytes expressing NK cell markers and T cell receptors [5]. NKT cells have two subtypes: invariant NKT cells (iNKT) and variant NKT cells (vNKT) [6]. iNKT cells are abundant in liver tissue and can be activated by various antigens including glycolipid and cytokines such as IL‐18 and TNF‐α [7, 8]. Activated iNKT cells can further stimulate other immune cells such as T cells and B cells, secrete cytokines and chemokines, and recruit neutrophils and monocytes to the liver [9, 10]. Studies have reported that CD1d^−/−^ mice, which lack both iNKT cells and vNKT cells, are resistant to halothane‐induced liver injury by regulating neutrophil recruitment [11]. However, other studies have shown that Jα18^−/−^ mice, which lack only iNKT cells, were susceptible to carbon tetrachloride‐induced acute liver injury via suppression of the proinflammatory effect of activated hepatic stellate cells [12]. Many studies have revealed that iNKT cells play promoting or inhibiting roles in different liver pathological states such as steatosis, carcinoma and HBV infection [13, 14, 15]. Most of these studies have described the function of iNKT cells from the molecular signalling pathway, which complicates experimental studies; iNKT cells can regulate the activity of immune cells, and liver tissues are rich in various immune cells such as Kupffer cells and natural killer (NK) cells. Therefore, observing the interaction between iNKT cells and other immune cells may help to clarify the function of iNKT cells in AILI.

Kupffer cells (KCs), as liver resident macrophages, reside within the lumen of the liver sinusoids and play a critical role in various liver diseases. Together with other immune cells, they form the first innate immune defence against bacteria and endotoxin entering the portal vein through the gastrointestinal tract [16]. Studies have revealed that early exposure to APAP can activate KCs in liver tissue, and the supernatant of APAP‐treated hepatocytes can activate a murine macrophage cell line [17]. In addition, some studies have shown that after APAP induction, KCs experience a process of reduction and self‐renewal [18]. These findings further demonstrate the important role of KCs in the development of AILI.

Although both iNKT and KCs play important roles in drug‐induced liver injury, little research has been conducted on the dynamic interaction between iNKT and KCs in the development of liver injury. In our previous study, we found that iNKT cells interact with KCs to clear free lipids during steatohepatitis [19]. In future research, we will further explore the role and mechanism of iNKT cells in AILI, along with the dynamic interactions among immune cells.

## 2 Materials and Methods

### 2.1 Mice

Cxcr6^*Gfp/+*^ mice and Jα18^−/−^ mice with a C57BL/6J background aged 8–10 weeks (19–25 g) were provided by Dr. Zhigang Tian (University of Science and Technology, School of Life Science, Hefei, China), and CD1d^−/−^ mice with a C57BL/6J background aged 8–10 weeks were provided by Dr. Yongwen Chen (Army Medical University, Chongqing, China). All mice were housed in a pathogen‐free, double‐barrier unit at 22°C in the Animal Research Centre of Nanjing Medical University under a 12‐h light/dark cycle, with water and food provided *ad libitum*. All protocols were approved by the Nanjing Medical University Animal Care Committee (protocol #2103048) and conducted in accordance with the guidelines established by the China Council on the Use of Laboratory Animals.

### 2.2 Animal Treatment and Confocal Laser Scanning Microscopy

Male mice aged 8–10 weeks‐old were intraperitoneally (i.p.) [20] injected with APAP (200 mg/kg, Sigma‐Aldrich, St. Louis, MO) dissolved in warm phosphate‐buffered saline (PBS) after 12 h of overnight fasting.

Transgenic Cxcr6^Gfp/+^ mice were used to visualise hepatic iNKT cells [21]. Cxcr6^Gfp/+^ mice were injected with 200 μg anti‐asialo GM1 antibody via the tail vein to eliminate NK cells. Kupffer cells were labelled intravenously with 10 μg Alexa Fluor 647‐conjugated F4/80 antibodies (BioLegend, San Diego, CA, USA). Kupffer cell depletion was achieved by injecting 200 μL clodronate liposomes (CLL) (Yeasen Biotechnology, Shanghai, China) into the tail vein 24 h before APAP injection. Liver sinusoids were visualised by injecting 10 μg Alexa Fluor 647‐conjugated CD31 antibodies (BioLegend). To verify the function of IL‐12, it was blocked by intravenous injection of 100 μg of purified anti‐IL‐12 antibodies (BioLegend) or 100 μg IgG isotype (BioLegend) control 12 h before APAP treatment. CD86 molecular localisation was visualised by injecting 10 μg PE‐conjugated CD86 molecule antibody (BioLegend) [21, 22, 23].

The mice were anaesthetised with ketamine (100 mg/kg) and xylazine (10 mg/kg) intravenously. Preparation of the murine liver for intravital microscopy has been described previously [21, 22, 23]. Intravital microscopy was performed using an LSM880 multi‐photonaser‐scanning microscope (Zeiss, Oberkochen, Germany) equipped with an Olympus focus drive and motorised stage (Applied Scientific Instrumentation, Eugene, OR, USA).

### 2.3 Video Microscopy and Analysis

Intravital microscopy was performed using an LSM880 multi‐photon‐photon laser‐scanning microscope (Zeiss, Oberkochen, Germany) equipped with an Olympus focus drive and motorised stage (Applied Scientific Instrumentation, Eugene, OR, USA). Visualisation of iNKT cells and other immune cells in the liver vasculature was achieved with five laser‐excitation wavelengths in rapid succession (488, 561, and 633) and captured with appropriate band‐pass filters (Semrock and Chroma). For each experiment, a minimum of three fields was imaged per mouse over 1 h. A 20× objective lens was used to collect real‐time 2D images with an image resolution of 1024 × 1024 pixels and a frame interval of 30 s. Each video was 15 min long. Cell movements were analysed using Imaris 7.4.2 (Bitplane, Zurich, Switzerland). Cells were tracked based on their fluorescent properties using Imaris Cell, Imaris Track, and Imaris Measurement Pro. Cells and molecules were identified based on their fluorescent properties using Imaris Surfaces, and movies were produced using Imaris Video, using the tracking function of Image J software and the Chemotaxis and Migration Tool to draw a discrete map of cell motion trajectory. In addition, 3D static image acquisition still uses a 20× objective lens and the Z‐Stack mode for imaging, with a resolution of 1024 × 1024 pixels, a *Z*‐axis depth of 10–15 μm, and a frame interval of 1 μm.

### 2.4 Histology

Mice were anaesthetised with ketamine (100 mg/kg) and xylazine (10 mg/kg) intravenously, perfused with ice‐cold PBS, and liver tissues were harvested, embedded in paraffin, and sliced into 5‐μm thick sections. The sections were stained with haematoxylin and eosin (H&E). Infiltrated neutrophils were quantified using antibodies against Ly‐6G (Sigma‐Aldrich) and assessed by bright‐field microscopy and quantified using ImageJ (NIH).

### 2.5 Biochemical Assays

Mice were anaesthetised with ketamine (100 mg/kg) and xylazine (10 mg/kg) intravenously, perfused with ice‐cold PBS, and then liver tissues and sera were harvested. ALT, AST, MPO, and GSH activities were quantified using commercial kits (Jiancheng Tech, Nanjing, China) according to the manufacturer's instructions.

### 2.6 Flow Cytometry

The liver or spleen tissue was gently homogenised by grinding and mincing and then ground to form a single cell suspension. Mouse venous blood was collected from the inner canthal vein, and red blood cells were lysed on ice. Lymphocytes were purified by Percoll (Pharmacia, New York, NY, USA) gradient centrifugation. For GFP cells, iNKT cell populations were characterised by staining with allophycocyanin (APC)‐conjugated anti‐CD1d‐α‐Galcer (BioLegend) for 30 min at 4°C. Dead cells were excluded from the analysis by adding 1 μL of Zombie NIR (Cat #:423106, APC‐Cy7; BioLegend) 10 min before flow cytometry. The number of NKT cells in each liver sample was determined by flow cytometry. In addition, we used Treg cell flow cytometry antibody (BioLegend: FITC anti‐mouse CD4, Cat #:100405; PE anti‐mouse CD25, Cat #: 101903; Alexa Fluor 647 anti‐mouse FOXP3, Cat #: 126408) to mark Treg cells in liver tissue. The number of Treg cells in each liver sample was determined using flow cytometry. The FACS Calibur Cell Analyser (Becton Dickinson, Franklin Lakes, NJ, USA) and FlowJo software (version 10.0.7; NIH, Bethesda, MD, USA) for data analysis.

### 2.7 Enzyme‐Linked Immunosorbent Assay

The iNKT cells in the liver were sorted by CD1d‐α‐Galcer tetramers (BioLegend) and cultured for 4 h. After 6 h of stimulation with PMA (MCE, Cat #: HY‐18739), iNKT cells were collected. The quantitative measurement of IL‐4 and TNF‐γ was using the Enzyme‐Linked Immunosorbent Assay (ELISA) Kit (Cat #: ab100710, Cat #: ab282874), following the manufacturer's protocol. OD values were measured at 450 nm using a microplate reader (Bio‐Tek, Winooski, VT, USA).

### 2.8 Liver Frozen Sections and Immunofluorescence of NETs

The liver tissue was trimmed and embedded in OCT cryoembedding medium. After freezing in a −80°C refrigerator, a Leica cryostat was used to make frozen sections with a thickness of 8 μm. The sliced tissues were adsorbed on positively charged anti‐shedding slides in sequence. After the frozen sections were naturally dried for 30 min, they were fixed with 4% PFA (paraformaldehyde) for 30 min, then rinsed with DPBS three times, 5 min each time, and permeabilized with 1% Triton X‐100 for 20 min. Next, 250 μL of each mixture of 3% BSA and 10% goat serum was dripped into the frozen sections and incubated at room temperature for 1 h. The incubation solution was gently poured off and rinsed with DPBS again 3 times for 5 min each time, and then 100 μL of primary antibody dilution solution (APC anti‐mouse Ly‐6G, Cat #: 108412, BioLegend; Rabbit anti mouse NE, Abcam, Cat #: ab310335; Goat anti mouse MPO, Cat #: AF3667) was added. The sections were incubated in a wet box at 4°C overnight, rinsed with DPBS three times, 5 min each time, AF488 and AF594 secondary antibodies and DAPI were added, and the sections were incubated in a wet box at 4°C for 1 h in the dark. The sections were rinsed with DPBS twice, 15 min each time, and finally, the sections were sealed with an anti‐fluorescence quencher and photographed in the dark room with a Zeiss inverted visualisation fluorescence microscope.

### 2.9 Real‐Time RT‐PCR

Liver tissues and iNKT cells were homogenised using TRIzol reagent (Takara Bio, Shiga, Japan). Total RNA was reverse transcribed into cDNA using SuperScript II reverse transcriptase (Thermo Fisher Scientific). Real‐time polymerase chain reaction (PCR) was performed using the IQ SYBR Green SuperMix Reagent (Bio‐Rad Laboratories, Hercules, CA, USA) on a real‐time PCR machine (CFX96, Bio‐Rad, USA) according to the manufacturer's instructions. The changes in gene expression were calculated using the comparative Ct method. For quantification, the values were analysed by normalising to the GAPDH mRNA expression. Primers used are listed in Table S1.

### 2.10 Data Processing

RNA sequencing data of patients with AILI and normal individuals were downloaded from the GEO database (accession: GSE74000). A heatmap was plotted by https://www.bioinformatics.com.cn (last accessed on 20 June 2024), an online platform for data analysis and visualisation.

### 2.11 Statistical Analysis

Intravital single‐photon laser scanning microscopy (SPLSM) data were analysed using Imaris 7.4.2 (Bitplane). Cells and molecules were identified based on their fluorescence properties using Imaris Surfaces, and movies were produced using Imaris Video. We used the tracking function of ImageJ software and the Chemotaxis and Migration Tool to draw a discrete map of the cell motion trajectory. All data were expressed as the means ± SEM. Data were compared using an unpaired two‐tailed Student's *t*‐test or one‐way analysis of variance (ANOVA) using GraphPad Prism software (version 5.02; GraphPad Software Inc., La Jolla, CA, USA). Bonferroni's multiple comparisons test was performed using SPSS (version 17.0 for Windows; IBM, Armonk, NY, USA).

## 3 Results

### 3.1 iNKT Deficiency Results in Aggravated Liver Injury in Mice

**FIGURE 1:**
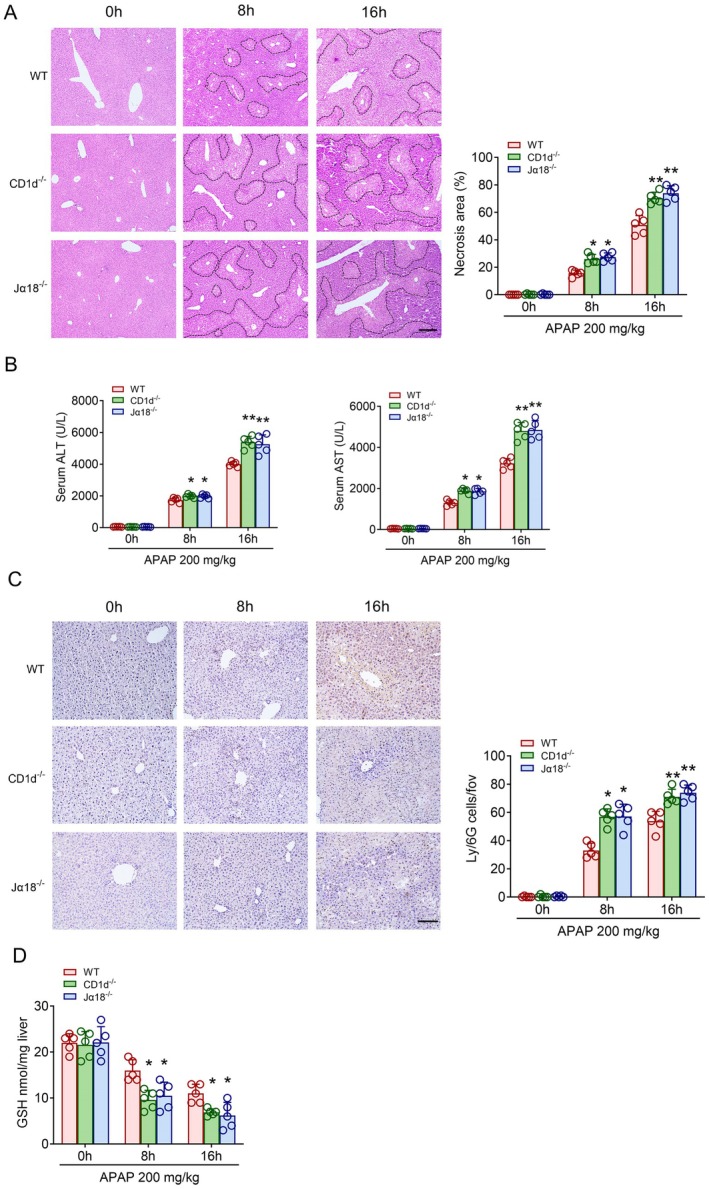
NKT deficiency aggravated APAP‐induced liver injury. (A) H&E staining and necrotic area quantification of liver tissues from Cxcr6^Gfp/+^, CD1d^−/−^ and Jα18^−/−^ mice after 0, 8, 16 h of APAP treatment, scale bar: 200 μm. (B) Serum ALT and AST levels from Cxcr6^Gfp/+^, CD1d^−/−^ and Jα18^−/−^ mice after 0, 8, 16 h of APAP treatment (*n* = 5). (C) Infiltrated Ly‐6G positive cells and cell number quantification from Cxcr6^Gfp/+^, CD1d^−/−^ and Jα18^−/−^ mice after 0, 8, 16 h of APAP treatment, Scale bar: 100 μm. (D) GSH level quantification from Cxcr6^Gfp/+^, CD1d^−/−^ and Jα18^−/−^ mice after 0, 8, 16 h of APAP treatment. Data are presented as relative expression ± SEM. *, *p* < 0.05; **, *p* < 0.01; using a two‐tailed unpaired Student *t* test.

Previous studies have shown that NKT deficient mice are more susceptible to DILI [24]. Firstly, we compared the degree of liver damage in wild type (WT) mice, CD1d^−/−^ mice, and Jα18^−/−^ mice induced by excessive APAP, and found that the liver damage of CD1d^−/−^ mice and Jα18^−/−^ mice was significantly higher than that in WT mice (Figure 1A). With the aggravated liver injury, serum levels of ALT and AST were also significantly increased. However, there was no significant difference between CD1d^−/−^ and Jα18^−/−^ mice (Figure 1B). In addition, we also found that CD1d^−/−^ and Jα18^−/−^ mice were more sensitive to APAP than WT mice in the mRNA expression of IL‐1β, IL‐6 and TNFα in liver tissue (Figure S1A). In addition, the level of myeloperoxidase (MPO), which can reflect the degree of tissue inflammation [25], was significantly increased in CD1d^−/−^ and Jα18^−/−^ mice liver after excessive APAP treatment (Figure S1B). We performed immunohistochemistry to observe the recruitment of neutrophils in the liver tissues and found that the number of neutrophils in CD1d^−/−^ and Jα18^−/−^ mice was significantly increased after APAP treatment (Figure 1C). However, flow cytometry showed that Treg cells were not significantly recruited to the liver after 16 h of APAP treatment in WT, CD1d^−/−^ and Jα18^−/−^ mice(Figure S2A). We also tested the changes in glutathione levels in the livers of different mice and found that the glutathione levels in the livers of CD1d^−/−^ and Jα18^−/−^ mice were significantly decreased after APAP treatment (Figure 1D). In addition, to clarify whether there are changes in NKT cells in liver tissue after APAP use in humans, we searched the gene expression omnibus (GEO) database for liver biopsy specimens from patients with AILI and healthy controls. By gene set enrichment analysis (GSEA), we surprisingly found that there were significant differences in the enrichment profiles between NKT cells and hepatocytes (Figure S3A) [26]. These results indicate that iNKT cells play an important role in the development of APAP‐induced drug‐induced liver injury and that iNKT deficient mice are more susceptible to APAP‐induced liver injury.

### 3.2 APAP Induced Activation of the Recruitment iNKT Cells With Dynamic Changes

**FIGURE 2:**
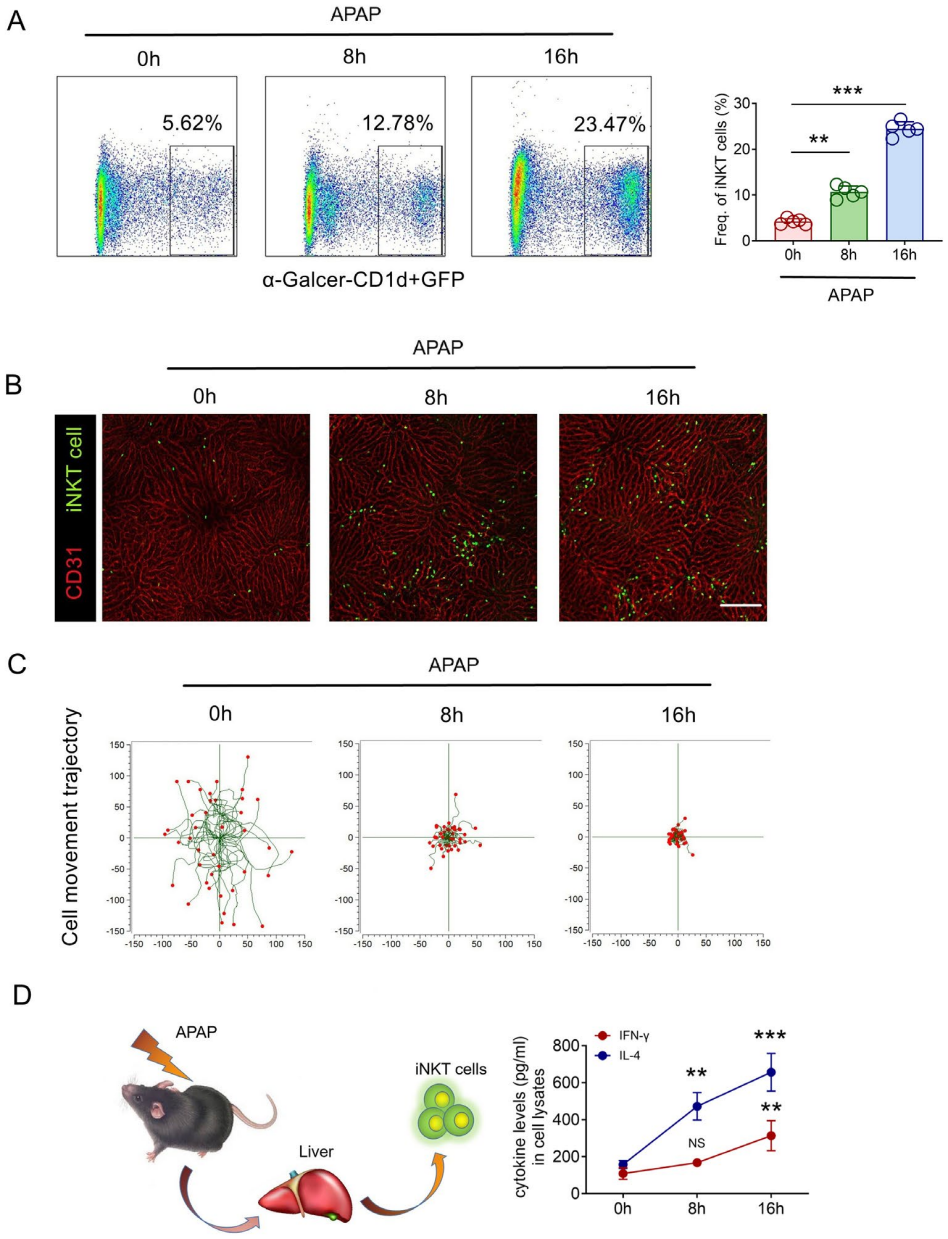
APAP induced iNKT recruitment and activation with dynamic changes. (A) Flow cytometry evaluated frequencies of iNKT cells among liver leukocytes of Cxcr6^Gfp/+^ mice (*n* = 5) after 0, 8, and 16 h of APAP treatment and quantification of recruited iNKT cells. (B, C) Liver tissues of Cxcr6^Gfp/+^ mice treated with APAP for 0, 8, and 16 h were observed by intravital microscopy, scale bar: 200 μm. Aligning starting positions of iNKT cells and plotting their migratory tracks. Each plot was collected from 40 iNKT cells from three different Cxcr6^Gfp/+^ mice, and each scan was imaged for 15 min. (D) Quantitative measurement of IFN‐γ and IL‐4 in iNKT cells in Cxcr6^Gfp/+^ mice (*n* = 5) after 0, 8, and 16 h of APAP treatment using ELISA kits and quantification of cytokine expression ratios. Data are presented as relative expression ± SEM. **, *p* < 0.01; ***, *p* < 0.001 using a two‐tailed unpaired Student *t* test.

Previous studies have reported that iNKT cells are recruited to the liver after excessive APAP treatment [26]. Flow cytometry data also confirmed this phenomenon. Flow cytometry analysis of the leukocyte population showed that a large number of iNKT cells were recruited into the liver after APAP treatment (Figure 2A). To study the dynamic recruitment characteristics of iNKT cells in AILI, we used Cxcr6^*Gfp/+*^ transgenic mice, which have been widely used to study the behaviour and function of iNKT cells in vivo. Under normal conditions, the iNKT cells of Cxcr6^*Gfp/+*^ mice were evenly distributed in liver tissue and randomly patrolled in the sinuses (Video S1). Enhanced iNKT cell recruitment was observed at 8 h after APAP treatment, and the number of recruited iNKT cells increased over time (Figure 2B and Video S2). We also found that under the induction of excessive APAP, 3D visualisation imaging showed that the recruited iNKT cells were transformed from the sinuses to the liver parenchyma, forming scattered or clustered recruitment features (Video S3). Interestingly, intravital imaging of the liver revealed limited movement of recruited iNKT cells under excessive APAP induction (Figure 2C). Previous studies have found that iNKT cells are activated by α‐GalCer and stop moving quickly in vitro [27]. Therefore, these data suggest that the migration of iNKT cells recruited by AILI was significantly restricted.

Previous studies have shown that iNKT cells in the quiescent phase are highly expressed at levels of IL‐4 and IFN‐γ mRNA. When iNKT cells are stimulated by α‐GalCer, they rapidly activate and produce large amounts of IL‐4 and IFN‐γ [28]. We cleared NK cells to eliminate this effect and sorted iNKT cells using flow cytometry after 8 or 16 h of APAP induction. ELISA showed that the expression of IL‐4 in iNKT cells was increased, but the expression of IFN‐γ was not significantly changed after 8 and 16 h of APAP induction (Figure 2D). These results indicate that iNKT cells recruited in AILI have restricted movement and are in an activated state with high expression of IL‐4.

### 3.3 Macrophages Undergo M1 Polarisation and Highly Express IL‐12 During AILI

**FIGURE 3:**
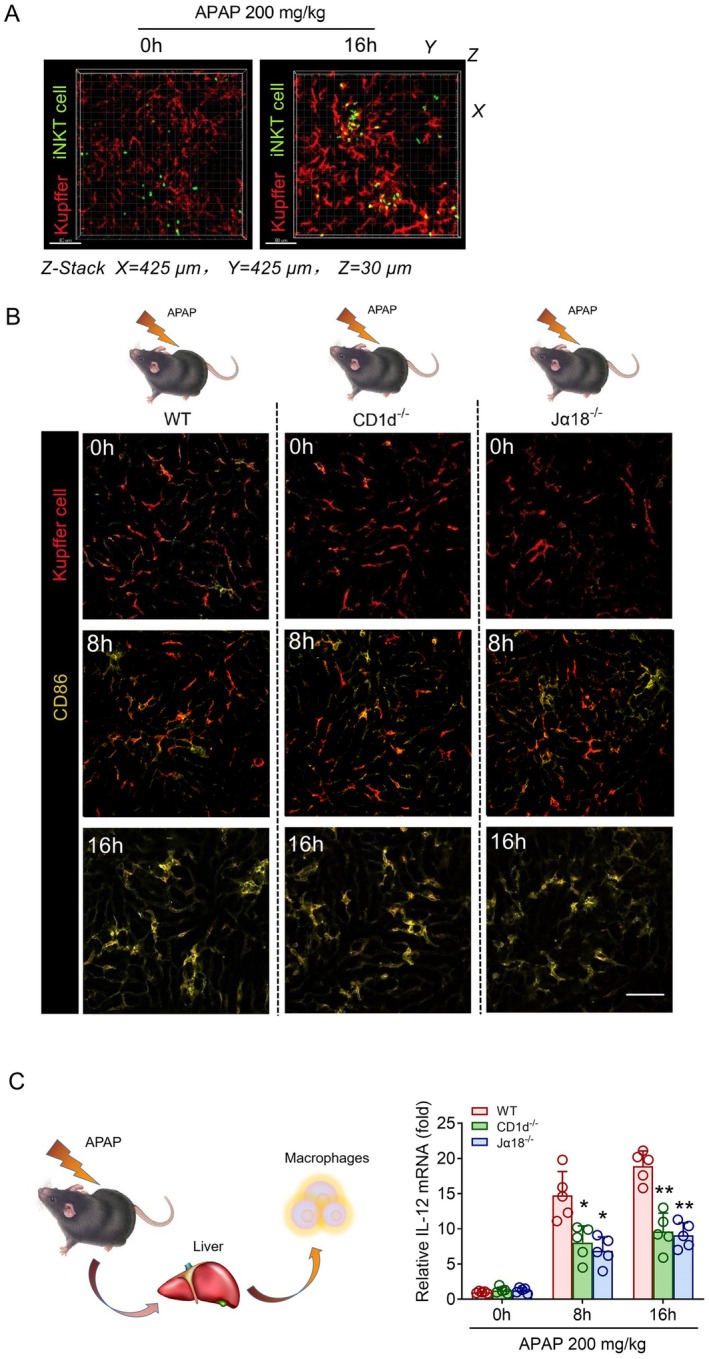
Kupffer cells interact with iNKT cells and undergo physiological changes during AILI. (A) Cxcr6^Gfp/+^ mice treated with APAP were subjected to hepatic intravital microscopy. Representative Z‐Stack images were obtained from 3 experiments performed to examine the interactions between iNKT cells (green) and Kupffer cells (fuchsia, labelled with Alexa Fluor 647‐conjugated anti‐mouse F4/80 antibodies). Scale bar: 100 μm. (B) Representative images were obtained from 3 independent experiments to examine the expression level of CD86 (yellow) in Kupffer cells (fuchsia, labelled with Alexa Fluor 647‐conjugated anti‐mouse F4/80 antibodies) from Cxcr6^Gfp/+^, CD1d^−/−^ and Jα18^−/−^ mice treated with APAP. Scale bar: 100 μm. (C) Relative mRNA levels of IL‐12 in Kupffer cells from Cxcr6^Gfp/+^, CD1d^−/−^ and Jα18^−/−^ mice treated with APAP (*n* = 5). Data are presented as relative expression ± SEM. *, *p* < 0.05; **, *p* < 0.01; using a two‐tailed unpaired Student *t* test.

As potential antigen‐presenting cells, Kupffer cells are involved in the activation of T lymphocytes in the liver [29]. Intravital imaging showed that Kupffer cells appeared to recruit and aggregate in the recruitment sites of iNKT cells and that these aggregated Kupffer cells interacted with iNKT cells dynamically (Figure 3A and Video S4). We also identified the characteristics of the interaction between iNKT cells and Kupffer cells using 3D imaging. We conducted a co‐localisation analysis on the contact surfaces of the two cell types and found significant interactions at the recruitment site (Video S5). In addition, we analysed the potential polarisation phenotype of the aggregated Kupffer cells. After APAP treatment, we found that both WT mice and NKT‐deficient mice had high expression of CD86 in the liver macrophages, and they were polarised to the M1 type as time progressed (Figure 3B). Next, Kupffer cells were sorted by flow cytometry to quantify IL‐12 mRNA levels in different groups. The results showed that compared with the control group, the Kupffer cells induced by APAP highly expressed IL‐12. And CD1d^−/−^ and Jα18^−/−^ mice induced by APAP also showed M1 polarisation and high IL‐12 expression. However, the ability of Kupffer cells to express IL‐12 in WT mice was significantly higher than that in CD1d^−/−^ and Jα18^−/−^ mice (Figure 3C). The above data indicated that Kupffer cells in AILI produced M1 polarisation and high IL‐12 expression.

### 3.4 Depletion of Macrophages Changes the Activation State of iNKT Cells and Aggravates AILI

**FIGURE 4:**
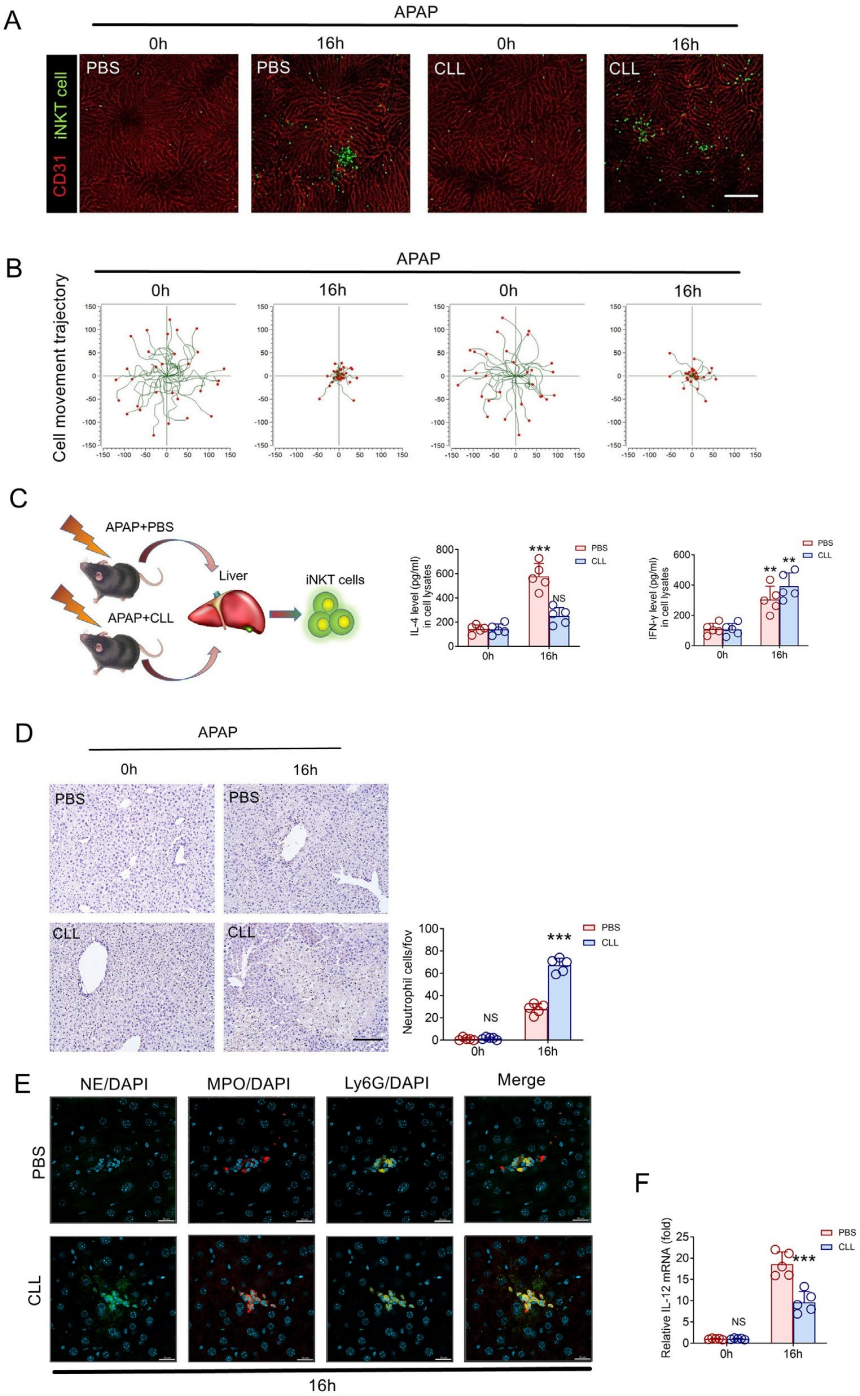
Interaction between Kupffer and iNKT cells reduces the degree of AILI. (A) Representative images were obtained from 3 independent experiments to examine iNKT cell response (green) to APAP and CLL. The sinusoidal endothelium was labelled using Alexa Fluor 647‐conjugated anti‐platelet endothelial cell adhesion molecule (CD31) antibodies (fuchsia). Scale bar, 200 μm. (B) iNKT cell migratory tracks plotted after aligning their starting positions. Each plot was collected from 30 iNKT cells from three different Cxcr6^Gfp/+^ mice, and each scan was imaged for 15 min. (C) Quantitative measurement of IL‐4 and IFN‐γ in iNKT cells of Cxcr6^Gfp/+^ mice (*n* = 5) treatment with APAP and/or CLL, and quantification of cytokines expression ratios. (D) Infiltrated Ly‐6G positive cells and cell number quantification of iNKT cells from Cxcr6^Gfp/+^ mice (*n* = 5) treatment with APAP and/or CLL. Scale bar: 100 μm. (E) Representative images of infiltrated neutrophils forming NETs from Cxcr6^Gfp/+^ mice (*n* = 5) treated with APAP and/or CLL; blue DAPI marks the nucleus, red MPO, green NE, and yellow Ly6G, Scale bar: 20 μm. (F) Relative mRNA level of IL‐12 in the livers from Cxcr6^Gfp/+^ mice treated with APAP and CLL (*n* = 5). Data are presented as relative expression ± SEM. **, *p* < 0.01; ***, *p* < 0.001 using a two‐tailed unpaired Student *t* test.

Next, we assumed that the biological effect of the interaction between Kupffer cells and iNKT cells was beneficial for resisting AILI. Therefore, we used clodronate liposomes (CLL) to deplete macrophages in Cxcr6^*Gfp/+*^ mice. The results showed that iNKT cells were still recruited in the liver of mice induced by excessive APAP (Figure 4A), and there was no significant difference in motor behaviour and quantity after recruitment (Figure 4B). Interestingly, under this condition, iNKT cells up‐regulated the expression of IFN‐γ and down‐regulated the expression of IL‐4 (Figure 4C). In addition, we also compared the mice treated with CLL and PBS induced by excessive APAP, and found that the liver injury of CLL‐treated mice was aggravated (Figure S4A), serum ALT and AST levels were also significantly increased, and the glutathione level that antagonises APAP‐induced liver injury was significantly decreased after CLL treatment (Figure S4B,C). Besides, the recruitment and adhesion of neutrophils also increased significantly in CLL‐treated mice, and the recruited neutrophils formed NETs in the liver after CLL treatment (Figure 4D,E). In addition, we found that the expression of IL‐12 in the liver of CLL‐treated mice decreased significantly (Figure 4F). These data suggest that the interaction between Kupffer cells and iNKT cells plays an important role in inhibiting excessive APAP‐induced liver injury, and Kupffer cells are likely to regulate the activation of iNKT cells through IL‐12.

### 3.5 Blocking IL‐12 Aggravates the Degree of AILI

**FIGURE 5:**
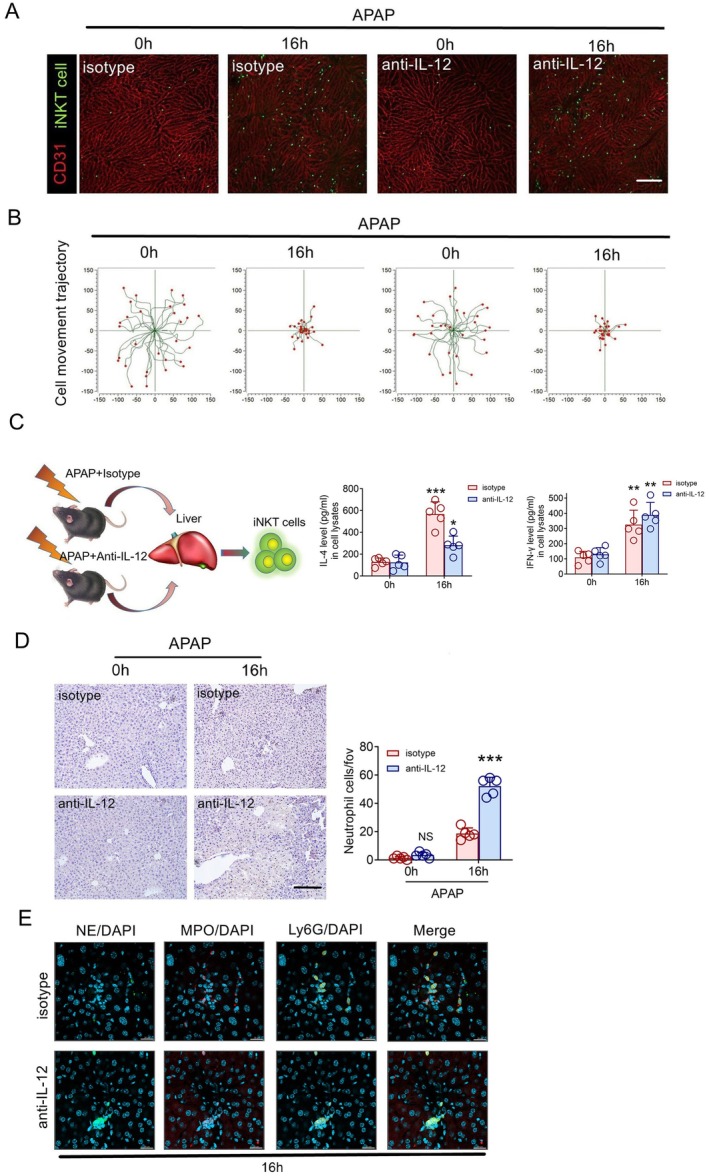
IL‐12 directly regulates the state of iNKT cells. (A) Representative images were obtained from 3 independent experiments to examine iNKT cell response (green) to APAP and anti‐IL‐12 antibody. The sinusoidal endothelium was labelled using Alexa Fluor 647‐conjugated anti‐platelet endothelial cell adhesion molecule (CD31) antibodies (fuchsia). Scale bar, 200 μm. (B) iNKT cell migratory tracks plotted after aligning their starting positions. Each plot was collected from 30 iNKT cells from three different Cxcr6^Gfp/+^ mice, and each scan was imaged for 5 min. (C) Quantitative measurement of IL‐4 and IFN‐γ in iNKT cells of Cxcr6^Gfp/+^ mice (*n* = 5) treated with APAP and/or anti‐IL‐12 antibody using ELISA kits and quantification of cytokine expression ratios. (D) Infiltrated Ly‐6G positive cells and cell number quantification of iNKT cells from Cxcr6^Gfp/+^ mice (*n* = 5) treated with APAP and/or anti‐IL‐12 antibody. Scale bar: 100 μm. (E) Representative images of infiltrated neutrophils forming NETs from Cxcr6^Gfp/+^ mice (*n* = 5) treated with APAP and/or anti‐IL‐12 antibody. Blue DAPI marks the nucleus, red MPO, green NE, and yellow Ly6G, Scale bar: 20 μm. Data are presented as relative expression ± SEM. *, *p* < 0.05; **, *p* < 0.01; ***, *p* < 0.001 using a two‐tailed unpaired Student's *t* test.

The above data show that Kupffer cells are likely to regulate the activation state of iNKT cells through IL‐12. Therefore, we speculated that IL‐12 is a cytokine that directly regulates the state of iNKT cells. We injected purified anti‐IL‐12 antibody into Cxcr6^*Gfp/+*^ mice through the tail vein, and then induced liver injury with excessive APAP. Through intravital imaging, we found that consistent with the results of CLL to deplete macrophages, blockade of IL‐12 did not inhibit the recruitment of iNKT cells (Figure 5A,B), but the degree of liver injury was aggravated, and we compared the mice treated with isotype and anti‐IL‐12 induced by excessive APAP. We found that the liver injury of anti‐IL‐12 treated mice was aggravated, and ALT and AST in serum were also significantly increased, and the glutathione level that antagonises APAP‐induced liver injury was significantly decreased after anti‐IL‐12 treatment (Figure S5A–C). After blocking IL‐12, the expression level of IL‐4 was downregulated significantly in iNKT cells, but the change of IFN‐γ was not very obvious (Figure 5C). In addition, we also observed increased recruitment and adhesion of neutrophils in IL‐12 antibody treated mice and the recruited neutrophils formed NETs in the liver after IL‐12 antibody treatment (Figure 5D,E). The above data showed that IL‐12 plays an important role in the process of anti‐AILI and can directly regulate the cytokines IL‐4 and IFN‐γ after activation of iNKT cells.

### 3.6 Adoptive Transfer of iNKT Cells Into Jα18^−/−^ Mice Alleviates AILI

**FIGURE 6:**
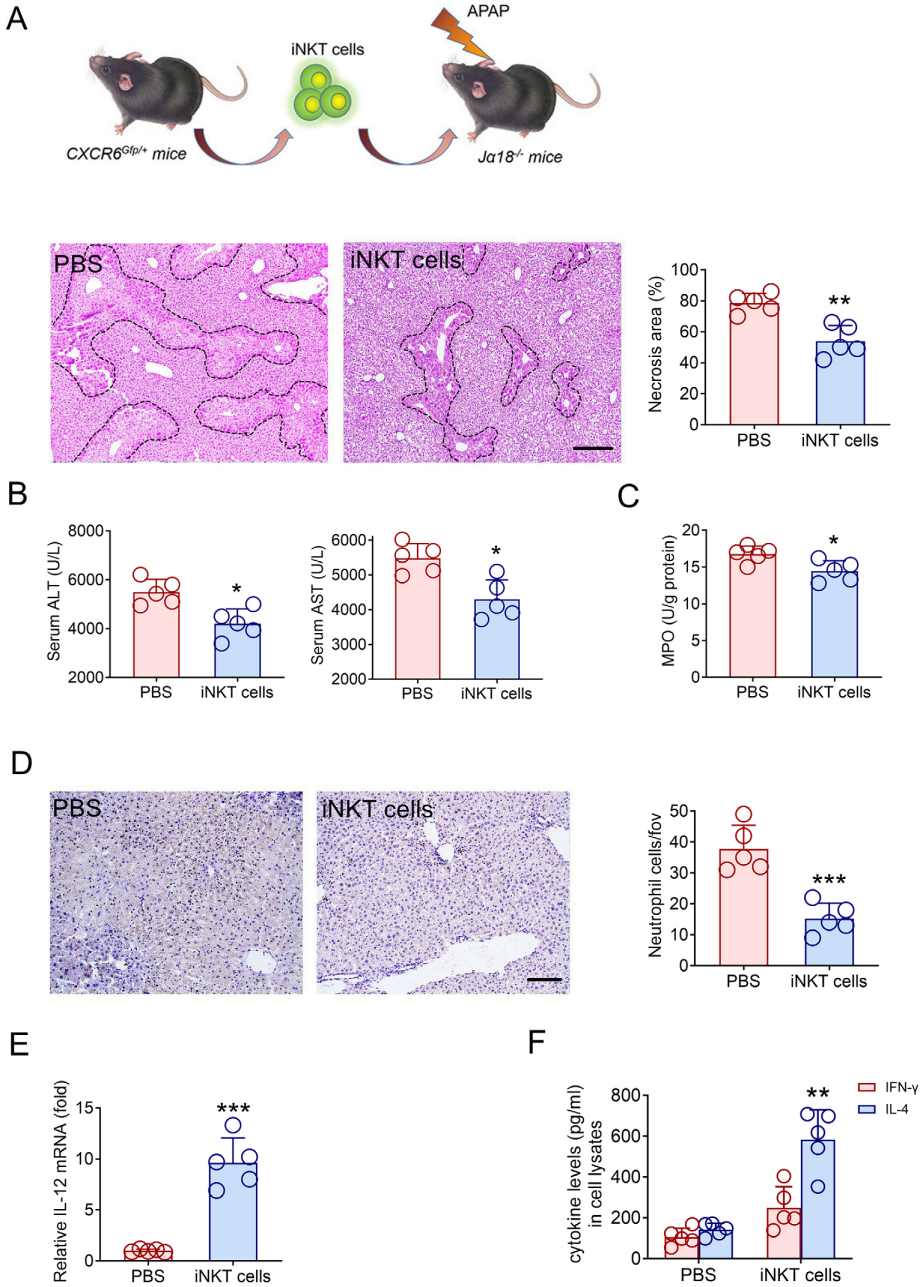
Adoptive transfer of iNKT cells alleviates AILI in Jα18^−/−^ mice. (A) H&E staining and necrotic area quantification of liver tissues from Jα18^−/−^ mice doptive transfer of PBS or iNKT cells after16h of APAP treatment, scale bar: 200 μm. (B) Serum ALT and AST levels from Jα18^−/−^ mice adoptively transferred with PBS or iNKT cells after 16 h of APAP treatment (*n* = 5). (C) Measurement of MPO activity in the liver tissues from Jα18^−/−^ mice adoptively transferred with PBS or iNKT cells after 16 h of APAP treatment (*n* = 5). (D) Infiltrated Ly‐6G positive cells and cell number quantification of Jα18^−/−^ mice liver adoptive transfer of PBS or iNKT cells after 16 h of APAP treatment (*n* = 5), Scale bar: 100 μm. (E) Relative mRNA level of IL‐12 in the Jα18^−/−^ mice liver adoptively transferred with PBS or iNKT cells after 16 h of APAP treatment (*n* = 5). (F) Quantitative measurement of IL‐4 and IFN‐γ in the Jα18^−/−^ mice liver adoptively transferred with PBS or iNKT cells after 16 h of APAP treatment using ELISA kits and quantification of cytokine expression ratios. Data are presented as relative expression ± SEM. *, *p* < 0.05; **, *p* < 0.01; ***, *p* < 0.001 using a two‐tailed unpaired Student *t* test.

From the above data, we know that IL‐12 produced by Kupffer cells can regulate the expression of IL‐4 and IFN‐γ after activation of iNKT cells, thereby resisting AILI. Therefore, we further verified whether the adoptive transfer of iNKT cells could alleviate the degree of AILI in Jα18^−/−^ mice. As expected, we found that the damage area of liver tissues (Figure 6A) and the transaminase in sera (Figure 6B) were significantly decreased in Jα18^−/−^ mice. In addition, the MPO and the number of neutrophil recruitment (Figure 6C,D) in liver tissues also showed a downward trend in Jα18^−/−^ mice.

In addition, we also found that after adoptive transfer of iNKT cells, Kupffer cells and iNKT cells exhibited dynamic interactive behaviour in the liver of Jα18^−/−^ mice (Figure S6A). Kupffer cells up‐regulated the expression of IL‐12 after adoptive transfer of iNKT cells (Figure 6E). We compared the levels of IL‐4 produced by iNKT cells before and after adoptive transfer, and found that iNKT cells recollected from APAP‐induced Jα18^−/−^ mice produced higher levels of IL‐4 (Figure 6F). The above data indicate that adoptive transfer of iNKT cells into Jα18^−/−^ mice can promote Kupffer cells to up‐regulate IL‐12, and thus positively feedback iNKT cells to produce more IFN‐γ, thereby inhibiting the inflammatory damage caused by the recruitment of neutrophils.

## 4 Discussion

Liver injury caused by the drug itself or its metabolites, or the reduction in the body's sensitivity or tolerance to drugs, is called drug‐induced liver injury (DILI) [30]. DILI can occur in healthy individuals without a history of liver disease or patients with serious diseases. Currently, there are more than 30,000 kinds of drugs and health products in our daily lives, and more than 1000 kinds of drugs can cause acute liver injury. Therefore, drug liver injury has become a serious public health problem that cannot be ignored [31]. Acetaminophen (APAP) is the most common drug that causes intrinsic liver injury and is the primary cause of acute liver failure in European and American countries [32].

Current research has predominantly concentrated on the hepatotoxic effects of APAP on hepatocytes. The liver, a major organ for innate and adaptive immunity, has a rich variety of immune cells that play multiple biological roles such as antigen presentation, cytokine production, and pro‐inflammatory effects. Through the analysis of liver biopsy samples from patients with AILI in the GEO database, we found that several types of immune cells in the liver tissue of patients with AILI, including NKT and Kupffer cells, exhibit significantly different enrichment patterns in their related signalling pathways compared to hepatocytes. Unlike hepatocytes, immune cells show a greatly increased mobility in the liver tissue. Current in vivo liver imaging technology reveals the spatiotemporal dynamic changes of APAP liver injury through multiple dimensions and levels, such as the recruitment of neutrophils, the ability of macrophages to capture bacteria, the regeneration ability of the liver after injury, and the evaluation of liver functional imaging, etc. [18, 33, 34, 35]; this not only accelerates the mechanism analysis (such as the causal relationship between liver injury and inflammation) but also provides a visualisation solution for the later evaluation of drug efficacy. Therefore, we used optical techniques to visually observe the movement trajectories of immune cells in the liver tissue and validated the interaction between NKT and Kupffer cells using molecular biology techniques, exploring new directions for the treatment of AILI.

Currently, research on the treatment of liver injury mostly adopts the time node slice or time node mechanism research scheme, and static research cannot fully reflect the panorama of the process of immune regulation after liver injury. As we all know, the biological functions of immune cells are inseparable from their microenvironment. The unique movement behaviour or morphology formed in the complex and changeable pathological microenvironment is not invariable, which also indicates that the biological functions of immune cells have dynamic spatio‐temporal characteristics. Therefore, using in vivo real‐time imaging technology to capture the micro details of the interaction between the same or multiple immune cells in the liver is more helpful in clarifying the immune regulatory mechanism in the pathogenesis of APAP‐induced liver injury.

NKT cells are mainly concentrated in the mouse liver, accounting for about 30% of liver lymphocytes [36], while iNKT cells are the most studied and well‐characterised NKT cells in mice and humans, expressing the Vα14 gene fragment and Jα18 gene fragment rearrangement αβT cell antigen receptor (TCR), and recognise glycolipid antigen presented by the non‐polymorphic MHC class I molecule CD1d. Studies have found that iNKT cells have the highest proportion in mouse liver and bone marrow, and studies have shown that iNKT cells play an important role in many diseases, such as fatty liver [19], liver ischaemia/reperfusion injury [37], liver virus infection, and liver cirrhosis [38]. Therefore, examining the role of iNKT cells in AILI using intravital imaging technology is important for a comprehensive understanding of the pathogenesis of liver disease and may provide insights for new treatments. Inflammatory mediators released by liver immune cells have been found to be related to the progression of APAP hepatotoxicity. NK and NKT cells play a key role in the progression of APAP‐induced liver injury by secreting IFN‐γ, regulating chemokine production, neutrophil accumulation, and upregulating FasL expression in the liver, all of which may promote the inflammatory response [39]. Some researchers have also found that neutrophil depletion can prevent the aggravation of APAP hepatotoxicity in mice [40]. In our study, we found that iNKT cells were recruited to the injury site and activated, releasing IL‐4 after AILI. The high expression of IL‐4 can inhibit the recruitment of neutrophils and reduce liver injury. We also found that the biological changes in iNKT cells are related to Kupffer cells.

Kupffer cells are macrophages that are fixed in the liver and located on the inner surface of hepatic sinuses. They can remove foreign antigens, antigen–antibody complexes, and cell fragments in the blood, which constitute an important part of the liver defence system [41]. It is found that Kupffer cells have both pro‐inflammatory and anti‐inflammatory effects in the process of liver injury induced by APAP and have an important impact on the outcome of liver injury [42]. Some studies have reported that the proinflammatory effects of Kupffer cells can exacerbate liver injury [17, 43]. However, other studies have shown that different administration methods of CLL can lead to different results, and studies have found that CLL pretreatment can exacerbate APAP‐induced liver injury [44, 45]. Similarly, in our study, we also found that CLL pretreatment exacerbated APAP‐induced liver injury. Considering the contradictory relationship between IL‐12 signalling and IFN‐γ dynamics in APAP‐induced hepatotoxicity observed in our studies, we speculate that the use of CLL or IL‐12 inhibitors during APAP‐induced liver injury will lead to a significant decrease in the levels of anti‐inflammatory cytokines such as IL‐10. In view of the complex interactions between cytokines in various models, we propose that the balance between pro‐inflammatory and anti‐inflammatory cytokines is crucial for regulating the immune response during APAP‐induced liver injury. Future studies should aim to clarify the exact mechanism of this cytokine crosstalk and its impact on the progression of liver injury, which is of utmost importance. In addition, Kupffer cells, as macrophages, have the function of phagocytising and cleaning up the surrounding necrotic tissue. However, when AILI causes extensive tissue damage, the phagocytic capacity soon reaches saturation and loses phagocytosis. Therefore, APAP causes the depletion of Kupffer cells in quantity and function, which may be one of the important factors that aggravate the degree of the inflammatory response. In our study, we confirmed that during AILI, macrophages experience M1 polarisation and highly express IL‐12. Kupffer cells regulate iNKT cell activation via IL‐12. We visually observed the interaction between iNKT cells and Kupffer cells by intravital imaging and clarified their mechanism in the occurrence and development of AILI by molecular biology technology.

In this study, although we have clarified that Kupffer cells induce iNKT cells to produce IL‐4 by IL‐12, thus inhibiting the recruitment of neutrophils in previous studies, Kupffer cells were mostly activated by DAMPs induced by APAP [46]. In addition to IL‐12, whether the activation of iNKT cells depends on DAMPs induced by hepatocyte necrosis is a biological event worthy of further study.

Our research involved the biological effects of the dynamic interaction between iNKT cells and Kupffer cells. As we all know, the dynamic interaction between cells is complex and changeable. Obviously, the biological effects of neutrophil recruitment that we focus on are only a small part of the complex effect network. Further studies are needed to fully elucidate the biological effects of the dynamic interaction between iNKT cells and Kupffer cells.

Immune cells are characterised by the expression of transcription factors and the cytokines they produce. Accordingly, iNKT cells can be subdivided into three subtypes: iNKT1 (T‐bet/IFN‐γ), iNKT2 (GATA‐3/IL‐4) and iNKT17 (Ror‐t/IL) In addition, other studies have shown that there are other functional subsets of iNKT cells in the surrounding tissues [47]. However, in our study, we did not conduct a more detailed subgroup analysis of recruited iNKT. Although our results show that Kupffer cells induce iNKT cells to produce IL‐4 by producing IL‐12, the recruited iNKT cell types tend to belong to the iNKT1 subtype. However, this does not mean that there are no other subtype groups in the recruited iNKT cells. Therefore, clarifying the proportion of recruited iNKT cell subtypes will be one of our future research plans.

In conclusion, our study clearly demonstrated the pathophysiological process of the interaction between iNKT cells and Kupffer cells in protecting the liver from injury after AILI through intravital imaging technology and deeply analysed the mechanism. This study provides a basis for elucidating the pathogenesis of AILI and for identifying effective therapeutic targets.

## Author Contributions

**Yinling Li:** conceptualization (equal), data curation (equal), formal analysis (equal), investigation (equal), resources (equal), software (equal), visualization (equal), writing – original draft (equal). **Shuang Wen:** data curation (lead), investigation (lead), methodology (lead), resources (supporting). **Jie Liu:** formal analysis (lead), investigation (lead). **Shuwei Li:** funding acquisition (supporting), investigation (supporting), writing – review and editing (supporting). **Junping Shi:** conceptualization (equal), project administration (equal), writing – original draft (equal), writing – review and editing (equal). **Haitao Wang:** conceptualization (lead), funding acquisition (lead), investigation (lead), methodology (lead), software (lead), supervision (lead), writing – original draft (lead).

## Conflicts of Interest

The authors declare no conflicts of interest.

## Supporting information


**Data S1:** Supporting Information.

**Video S1:** Movement patterns of hepatic iNKT cells in normal liver sinusoids. The video was recorded for 10 min. In untreated *Cxcr6* ^Gfp/+^ mice, intravital imaging showed that hepatic iNKT cells patrol freely in liver sinusoids (Green, iNKT cells).

**Video S2:** Movement patterns of hepatic iNKT cells in AILI. The video was recorded for 30 min. After treatment with APAP, iNKT cells recruited into injury sites in liver. Green, iNKT cells; Red, hepatic sinusoid (stained with Alexa Fluor 647‐conjugated CD31).

**Video S3:** Three‐dimensional reconstruction of iNKT cells exuded from liver sinusoids in AILI. After treatment with APAP, the recruited iNKT cells exuded from the hepatic sinusoids. 15 confocal Z planes were recorded every 2 μm and reconstructed into a movie. Green, iNKT cells; Red, hepatic sinusoid are rendered in 3D (stained with Alexa Fluor 647‐conjugated CD31).

**Video S4:** Interaction between iNKT cells and Kupffer cells in AILI. The video was recorded for 20 min. After treatment with APAP, iNKT cells and Kupffer cells recruited into injury sites and interacted. Green, iNKT cells; Red, Kupffer cells (stained with Alexa Fluor 647‐conjugated F4/80 antibodies).

**Video S5:** Three‐dimensional reconstruction of interaction between iNKT cells and Kupffer cells. After treatment with APAP, the recruited iNKT cells interact with Kupffer cells. 15 confocal Z planes were recorded every 2 μm and reconstructed into a movie. Green, iNKT cells; Red, Kupffer cells (stained with Alexa Fluor 647‐conjugated F4/80 antibodies); The three‐dimensionally rendered blue represents the area where iNKT interacts with Kupffer cells.

## Data Availability

The data that supports the findings of this study are available in the Supporting Information of this article.
