# Supplementary material for: Kupffer Cells Regulate iNKT Cells Through Il‐12 to Mitigate the Extent of APAP‐Induced Damage in the Liver
Source: J Cell Mol Med. 2025 Jul 2;29(13):e70549. doi: 10.1111/jcmm.70549 (PMC12217646; doi:10.1111/jcmm.70549)
Supplement: Supplementary file 1 — Data S1: Supporting Information. [file JCMM-29-e70549-s001.docx]

**SUPPLEMENTARY FIGURES**

**
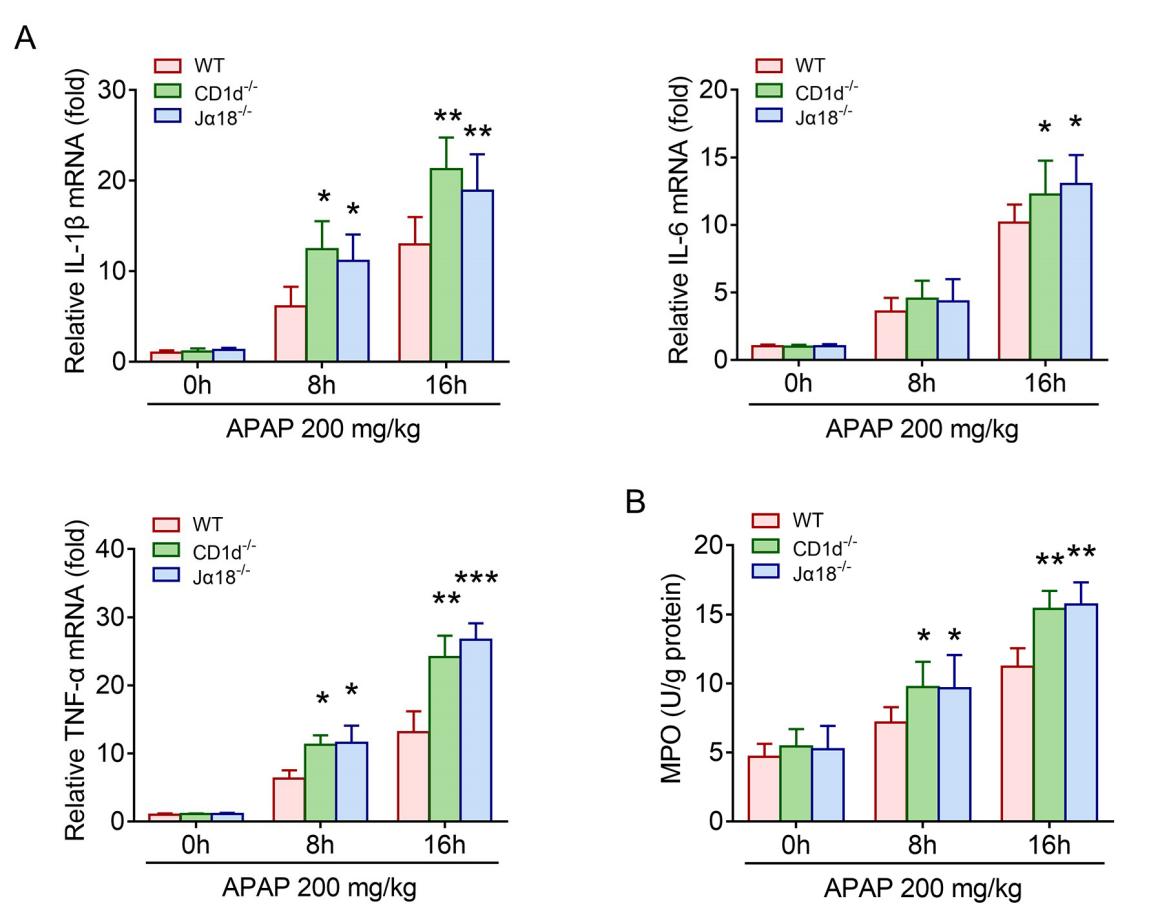
**

**Figure S1. NKT deficiency aggravated APAP-treated liver inflammation**

**A.** Relative mRNA level of IL-1β, IL-6 and TNF-α in the livers from Cxcr6^Gfp/+^, CD1d^-/-^ and Jα18^-/-^ mice after 0, 8, 16h of APAP treatment (n = 5). **B.** Measurement of MPO activity in the liver tissues from Cxcr6^Gfp/+^, CD1d^-/-^ and Jα18^-/-^ mice after 0, 8, 16h of APAP treatment (n = 5). Data are presented as relative expression ± SEM. *, *P* < 0.05; **, *P* < 0.01; ***, *P* < 0.001 using a two-tailed unpaired Student t test.


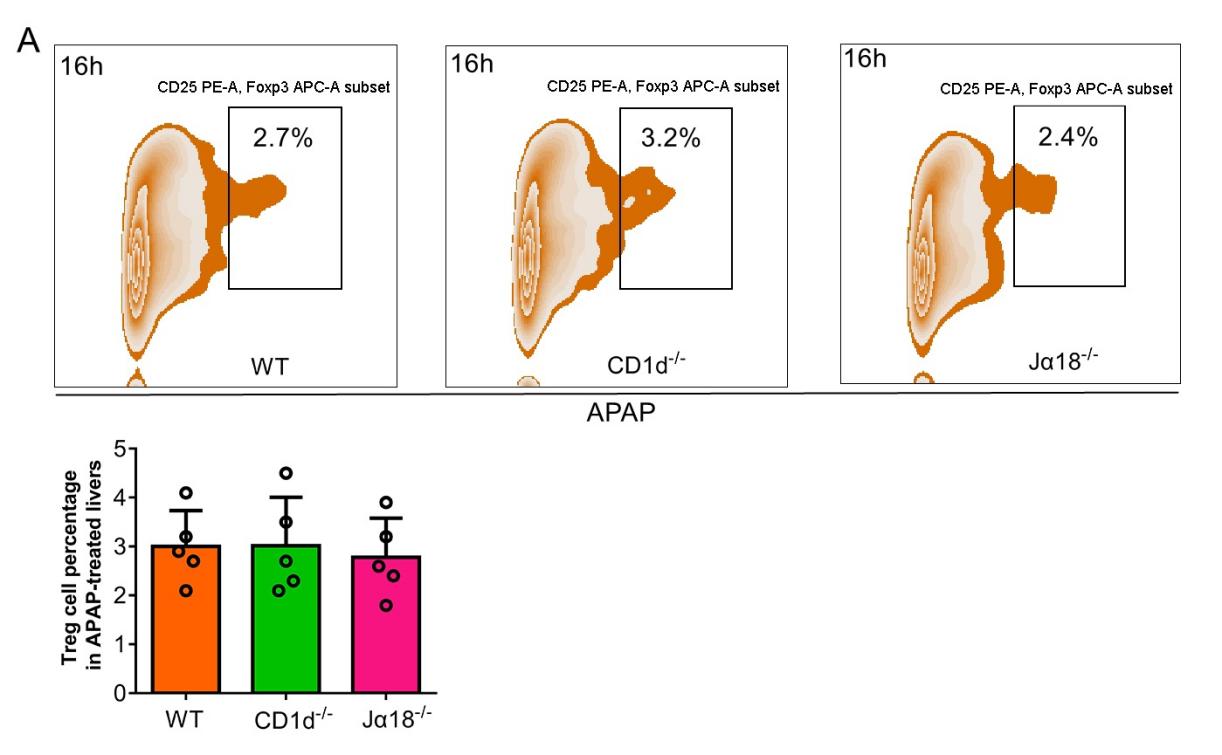


**Figure S2. Recruitment of Treg cells after APAP treatment in different mice**

A. Representative flow cytometry images of Treg cell recruitment after APAP treatment in different mice (n = 5). Data are presented as relative expression ± SEM. *, P < 0.05; **, P < 0.01; ***, P < 0.001 using a two-tailed unpaired Student t test.


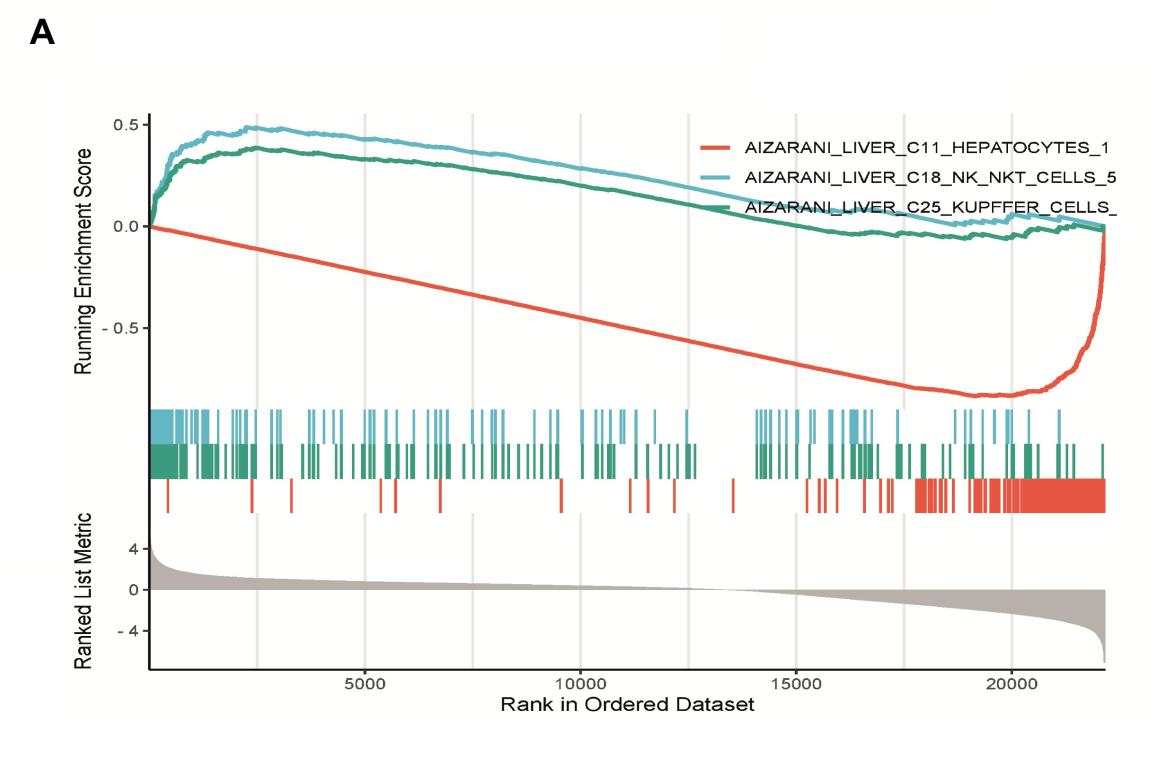


**Figure S3. Immune cells show different expression patterns in AILI**

A. The GSEA analysis of cell type signature gene sets between AILI patients and healthy controls revealed significant difference in immune cells and hepatocytes.


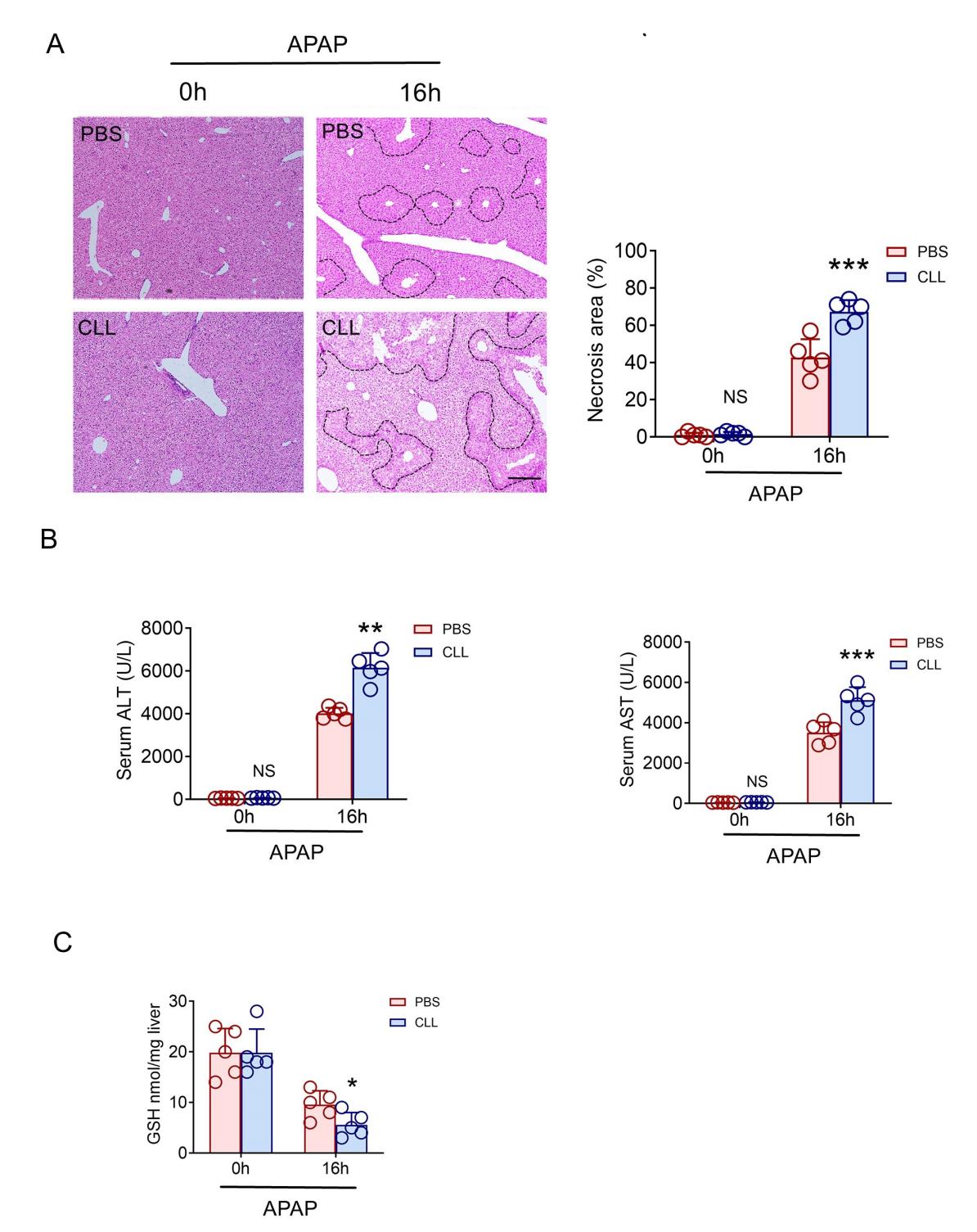


**Figure S4. Depletion of macrophages aggravate AILI**

**A.** HE staining and necrotic area quantification of liver tissues from Cxcr6^Gfp/+^ mice (n = 5) treated with APAP and / or CLL, scale bar: 200 μm. **B.** Serum ALT and AST levels from Cxcr6^Gfp/+^ mice (n = 5) treated with APAP and / or CLL. C. GSH levels from Cxcr6Gfp/+ mice (n = 5) treated with APAP and / or CLL. Data are presented as relative expression ± SEM. *, *P* < 0.05; **, *P* < 0.01; ***, *P* < 0.001 using a two-tailed unpaired Student t test.


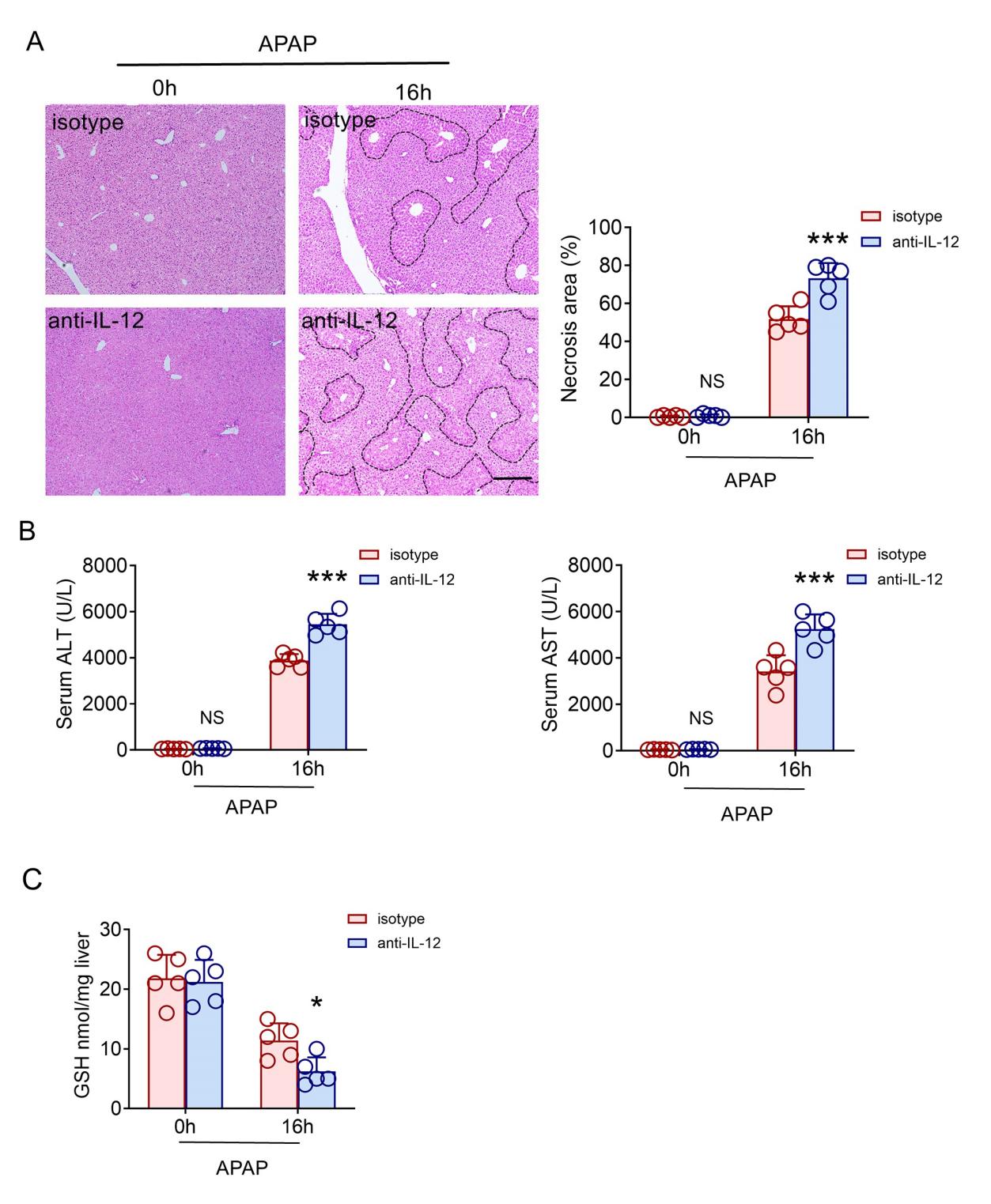


**Figure S5. Blocking IL-12 aggravate the degree of AILI**

**A.** HE staining and necrotic area quantification of liver tissues from Cxcr6^Gfp/+^ mice (n = 5) treated with APAP and / or anti-IL-12 antibody, scale bar: 200 μm. **B.** Serum ALT and AST levels from Cxcr6^Gfp/+^ mice (n = 5) treated with APAP and / or anti-IL-12 antibody.. C. GSH levels from Cxcr6Gfp/+ mice (n = 5) treated with APAP and / or anti-IL-12 antibody. Data are presented as relative expression ± SEM. *, *P* < 0.05; **, *P* < 0.01; ***, *P* < 0.001 using a two-tailed unpaired Student t test.

**
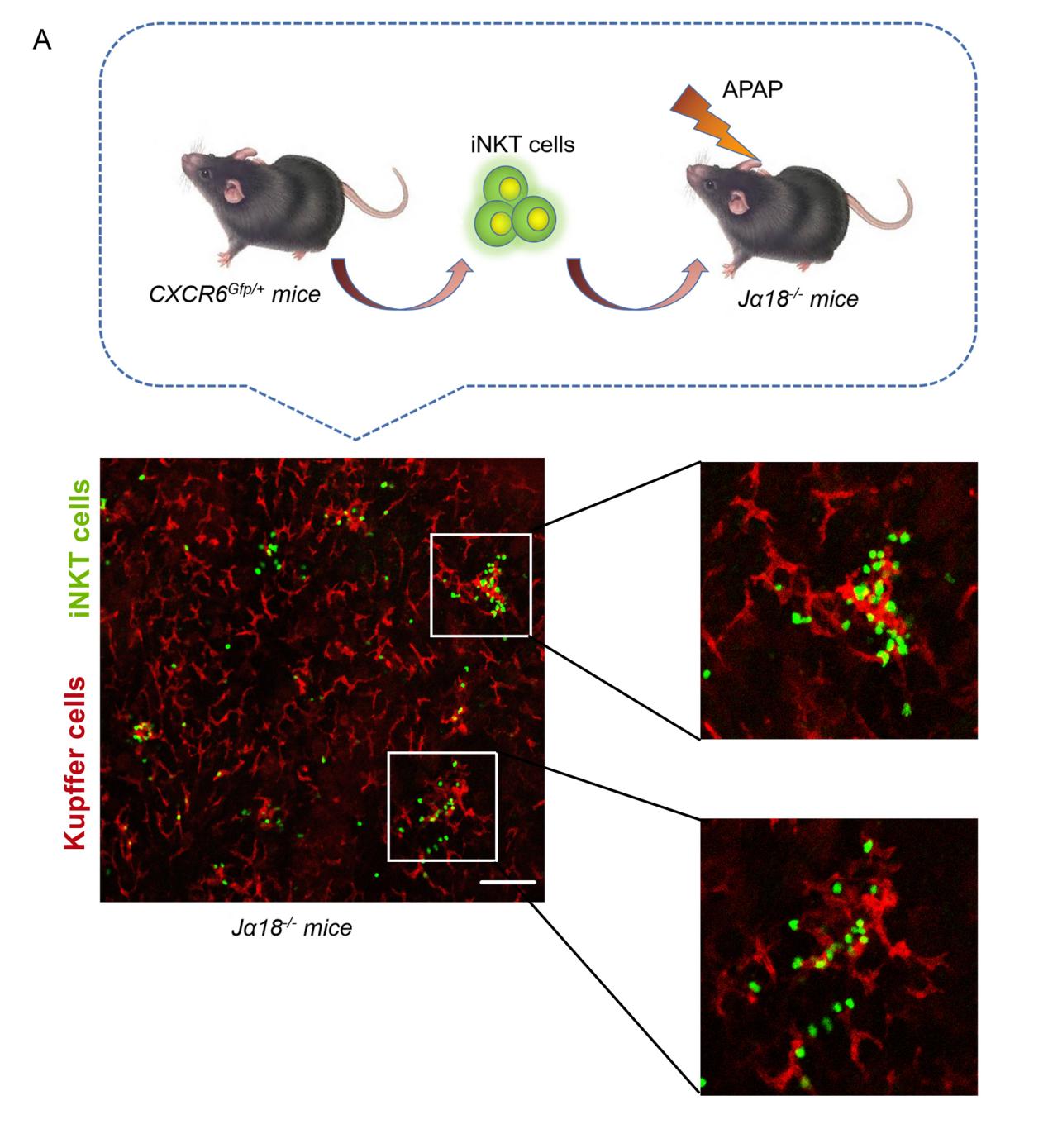
**

**Figure S6. Kupffer cells and adoptive transferred iNKT cells had dynamic interactive behavior**

**A.** Jα18^-/-^ mice adoptive transferred with iNKT cells were subjected to hepatic intravital microscopy. Representative Z-Stack images were obtained from 3 experiments performed to examine the interactions between iNKT cells (green) and Kupffer cells (fuchsia, labeled with Alexa Fluor® 647-conjugated anti-mouse F4/80 antibodies). cale bar: 100 μm.

**Video S1. Movement patterns of hepatic iNKT cells in normal liver sinusoids.**

The video was recorded for 10 min. In untreated *Cxcr6*^Gfp/+^ mice, intravital imaging showed that hepatic iNKT cells patrol freely in liver sinusoids (Green, iNKT cells).

**Video S2. Movement patterns of hepatic iNKT cells in AILI.**

The video was recorded for 30 min. After treatment with APAP, iNKT cells recruited into injury sites in liver. Green, iNKT cells; Red, hepatic sinusoid (stained with Alexa Fluor 647-conjugated CD31).

**Video S3. Three-dimensional reconstruction of iNKT cells exuded from liver sinusoids in AILI.**

After treatment with APAP, the recruited iNKT cells exuded from the hepatic sinusoids. 15 confocal Z planes were recorded every 2 μm and reconstructed into a movie. Green, iNKT cells; Red, hepatic sinusoid are rendered in 3D (stained with Alexa Fluor 647-conjugated CD31).

**Video S4. Interaction between iNKT cells and Kupffer cells in AILI.**

The video was recorded for 20 min. After treatment with APAP, iNKT cells and Kupffer cells recruited into injury sites and interacted. Green, iNKT cells; Red, Kupffer cells (stained with Alexa Fluor 647-conjugated F4/80 antibodies).

**Video S5. Three-dimensional reconstruction of interaction between iNKT cells and Kupffer cells.**

After treatment with APAP, the recruited iNKT cells interact with Kupffer cells. 15 confocal Z planes were recorded every 2 μm and reconstructed into a movie. Green, iNKT cells; Red, Kupffer cells (stained with Alexa Fluor 647-conjugated F4/80 antibodies); The three-dimensionally rendered blue represents the area where iNKT interacts with Kupffer cells.

**Supplemental Table 1. List of primers.**

| qPCR primers for mouse genes | | |
| --- | --- | --- |
| Genes | Sequences (5ʹ→3ʹ) | |
| IL-1β | Forward | TGTCTTGGCCGAGGACTAAGG |
|  | Reverse | TGGGCTGGACTGTTTCTAATGC |
| IL-6 | Forward | ACAACCACGGCCTTCCCTAC |
|  | Reverse | TCCACGATTTCCCAGAGAACA |
| IL-12 | Forward | ATGAGGAGCTGGCTTTGGTC |
|  | Reverse | TTGCATCCATTTGTGTGGCG |
| TNF-α | Forward | ACGGCATGGATCTCAAAGAC |
|  | Reverse | AGATAGCAAATCGGCTGACG |
| CXCL-16 | Forward | GGGCTTTGGACCCTTGTCTC |
|  | Reverse | AGTTCTGCTGTGTCGCTCTC |
| GAPDH | Forward | TGCAGTGGCAAAGTGGAGATT |
|  | Reverse | TCGCTCCTGGAAGATGGTGAT |
